# Examining resilience in Singapore in the face of COVID-19 community restrictions

**DOI:** 10.3389/fpsyg.2023.1082148

**Published:** 2023-11-29

**Authors:** Alyssa Yenyi Chan, Chuen Seng Tan, Felicia Jia Hui Chan, Alexius Matthias Sheng En Soh, Mark I-Cheng Chen, Zoe Jane-Lara Hildon

**Affiliations:** ^1^Saw Swee Hock School of Public Health, National University of Singapore and National University Health System, Singapore, Singapore; ^2^National Public Health and Epidemiology Unit, National Centre for Infectious Diseases, Singapore, Singapore

**Keywords:** resilience, COVID-19 community restrictions, protection, adaptive strategies, psychosocial adversity

## Abstract

**Introduction:**

To curb transmission of COVID-19, Singapore has experienced multiple, ongoing community restrictions. Gaining the ability to adapt and thrive under pressure will be key to addressing effects of these restrictions on mental health. To inform this, we examine the following research questions, (1) What typifies adversity related to living with on–off COVID-19 restrictions? (2) Who are the resilient? (3) How are negative effects of adversity attenuated?

**Methods:**

Participants were a part of the Strengthening Our Community’s Resilience Against Threats from Emerging Infections (SOCRATES) cohort, invited to participate in this survey either via email or text message. Using the community survey data (*N* = 1,364), analyses including Wilcoxon rank sum test and logistic regression were conducted.

**Results:**

Adversities are identified as circumstances associated with a significant increase in Hospital Anxiety and Depression Scale (HADS) scores. These are typified by having financial worries; experiencing heightened emotions and frequent crying; having “out of body” experiences; having to move frequently or not being able to settle into accommodation; and regularly feeling mistreated by someone close to you. Being resilient in the face of adversity was determined by HADS scores for depression and anxiety (dichotomized at the median) and characterized by overall better social relationships such as having harmonious living situations and solution-driven coping strategies, especially the ability to harness the belief that difficult situations can lead to growth.

**Discussion:**

In accordance with the Loads-Levers-Lifts model, results indicate that initiatives that increase access to identified protection, while minimizing exposure to known adversities where possible, will promote resilience under COVID-19 restrictions.

## 1 Introduction

Since the arrival of the novel coronavirus (COVID-19) in Singapore, the country has dealt with multiple waves of infection and community mitigation strategies, reconfiguring daily life and social cohesion within neighborhoods. For residents in the local community this has meant having to navigate the dynamic nature of infection and ensuing restrictions. Empirical studies on infectious disease outbreaks and other influenza pandemics (i.e., SARS, H1N1, MERS) have shown variability in adaptation strategies (Chew et al., 2020; Javed and Parveen, 2021; Kavčič et al., 2022).

As the COVID-19 pandemic developed, systematic reviews and community research studies across multiple settings have demonstrated growing mental health concerns, specifically the rising trend in depression and anxiety (Brooks et al., 2020; Xiong et al., 2020; Wong L. P. et al., 2021; Castellano-Tejedor et al., 2022) associated with periods of heightened restrictions. However, a finding of greater concern is the persistence of these in the long term (Brooks et al., 2020). For instance, even during later stages of the pandemic (i.e., September 2020), where restrictions were lessened, a recent study found that more than half the sampled population were experiencing symptoms related to either anxiety or depression (Wong L. P. et al., 2021).

In this paper we define mental health as a state of well-being where an individual capitalizes on their abilities to work productively and cope with daily stressors to contribute meaningfully to their community (World Health Organization, 2018). More broadly, health is conceptualized as relying on the ability to gain homeostasis, returning to levels of usual functioning be this physiological or psychosocial (Huber et al., 2016) when facing stressors or external threats to adaptation. We take the stance that such resilient processes are underpinned by having the ability to obtain and mobilize both internal and external protection in the face of concomitant adversity (Egeland et al., 1993; Yates et al., 2003; Hildon et al., 2018). These processes are exemplified in a recent study of healthcare workers facing the early disruptions and fears raised by the pandemic (Chan et al., 2022), further described below. The forthcoming quantitative analysis based on self-administered online survey data, takes a similar approach, seeking to identify what protects the community, enabling better than expected mental health outcomes as their exposure to adversity mounts.

## 2 Theoretical underpinnings

Resilience research on adaptation during COVID-19 (Vinkers et al., 2020; Xiao et al., 2020; Blanc et al., 2021; Zhang et al., 2022) has been considered in various ways. Proponents of this approach tend to build on the long-standing traditions of resilience studies originally based on at-risk children in Hawaii (Werner and Smith, 1982; Gallo et al., 2009; Keyes, 2009; Zhang et al., 2022). These early waves of research helped define resilience as being able to do better than expected, even flourish despite adversity (Werner and Smith, 1979). The following paper defines and operationalizes resilience, accordingly, building on several waves of resilience research (Richardson, 2002), and taking a broad multi-disciplinary, theory-based perspective. Current studies on resilient responses to the pandemic tend to focus on providing evidence and recommendations for stress reduction and enablement of resilient outcomes. Resilient processes and types of protection that kick-in when things are likely to be “really bad” have been less considered.

**Figure 1:**
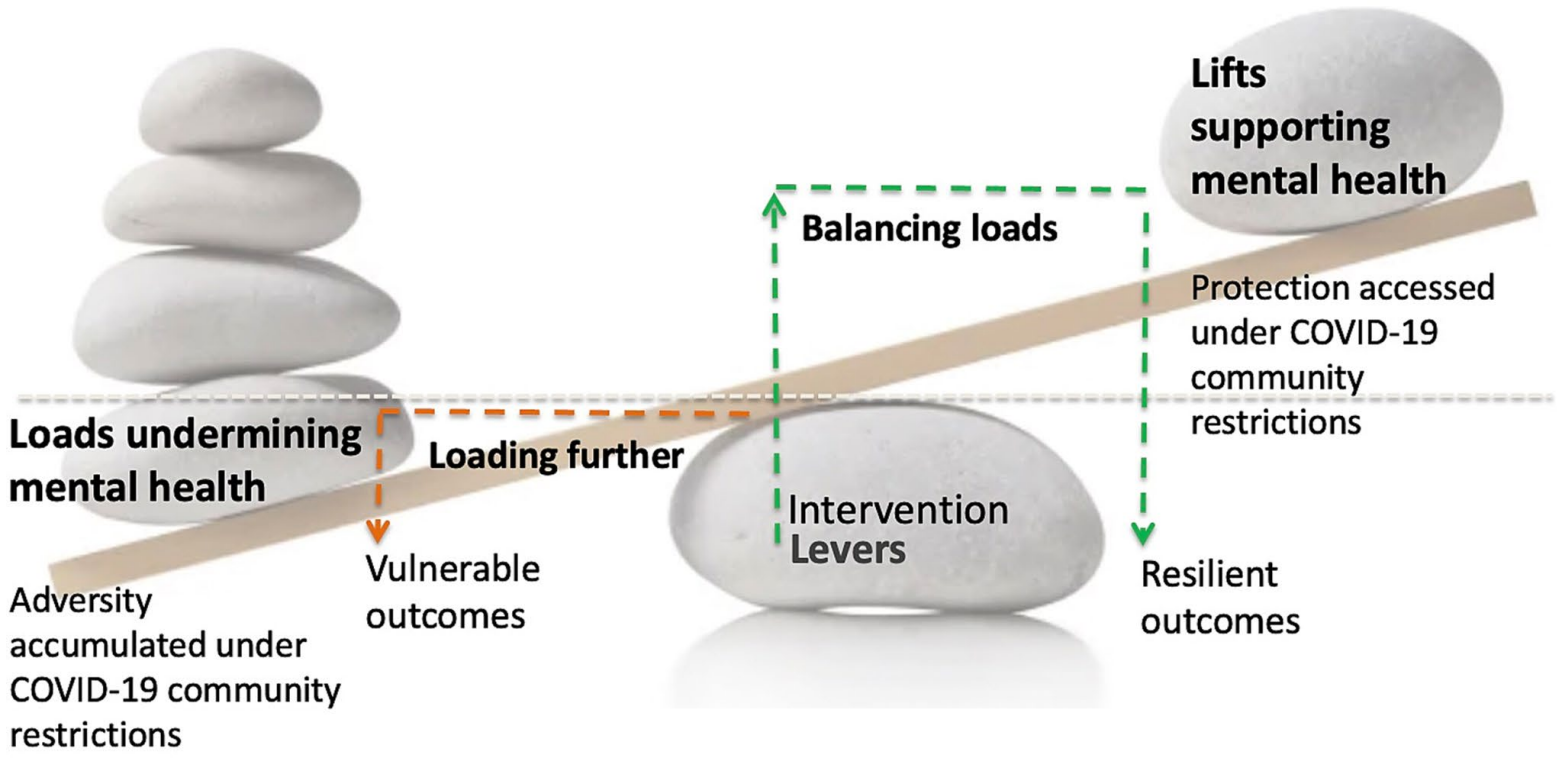
Loads-Levers-Lifts model for engineering resilience.

The Loads-Levers-Lifts model (Hildon et al., 2018; Chan et al., 2022) proposes resilience as a dynamic and modifiable process (see Figure 1). The process is conceived with adversity exposure as the starting point. Such exposure represents risk (i.e., loads), especially as these accumulate and become more challenging to overcome. In response, identifying protection that are needed to offset these effects (i.e., lifts), as well as the types of interventions (i.e., levers) to use will be central to understanding resilient mechanisms.

Therefore, resilience, though intuitive to some, or as Masten and colleagues have called it “ordinary magic” (Masten, 2001; Masten, 2015) is also a phenomenon that can be supported and engineered. This can be achieved by reducing loads or leveraging lifts. Identifying what these are and when lifts “kick in” when loads are high is the purpose of our forthcoming analyses.

## 3 Study aim and research question

Accordingly, we aim to understand how to adapt and thrive under multiple, potentially ongoing, community restrictions and adversities experienced during COVID-19 to inform their effects on the wider community’s mental health. More specifically, the study objectives are to identify:

1. Psychosocial, emotional and socio-environmental factors that contribute to adverse effects on mental health outcomes;
2. Resilient characteristics among those doing better than expected despite exposure to high adversity;
3. Attributes that attenuate the effects of high levels of adversity.

In summary, we examine three related research question – (1) What typifies adversity related to living with on–off COVID-19 restrictions? (2) Who are the resilient? (3) How are negative effects of adversity attenuated?

## 4 Methods

### 4.1 Study context and sampling approach

These data were collected via a self-administered survey, delivered through an online platform (FormSG), and rolled out as part of the Strengthening Our Community’s Resilience Against Threats from Emerging Infections (SOCRATES) cohort study. The cohort’s conception and full sampling strategy is detailed elsewhere (Lim et al., 2021). Recruitment was quasi-randomized, supplemented by community outreach. Our study was approved by the National Healthcare Group Domain Specific Review Board (DSRB) 2018/01203.

Analyses are based on data collected from 7th to 14th September 2021, from 1,364 out of 1,407 potential respondents who completed the questionnaire (item nonresponse and excluded cases *N* = 43). Supplementary Annex 1 summarizes community restrictions related to COVID-19 mitigation over time (see Infographic S1). We focused on the period just after May 2021 to July 2021, locally termed as Phase 2 Heightened Alert, where Singapore faced a resurgence of cases and tightening of community mitigation measures. Restriction fatigue kicked in, leaving some struggling to cope with what was perceived as a regression in progress against the pandemic (Sim et al., 2023).

### 4.2 Measures

#### 4.2.1 Mental health – hospital anxiety and depression scale (HADS)

Mental health was measured using a validated 14-item scale: Hospital Anxiety and Depression Scale (HADS; Zigmond and Snaith, 1983; Spinhoven et al., 1997; Singer et al., 2009). The measure has 7 items each for the two components, depression and anxiety, where each item is scored from 0 to 3 using a 4-point Likert scale, producing a range of 0 to 21 for each domain. A higher score indicates the presence of anxious or depressive symptoms.

#### 4.2.2 Psychosocial adversity

In this paper we set out to examine ways of redressing the effects of adversity exposures that are demonstrated to significantly worsen, and result in likely measurably “bad,” or worse than expected, mental health outcomes. We examined five selected adversities. See the results section for detailed breakdown of these. Broadly speaking these spanned financial and psycho-emotional domains of functioning and related experiences seen as potentially contributing to poorer mental health. These adversities were captured based on a tailored list of potential COVID-19 stressors faced after repeated lock downs, and were assessed using true/false responses. All adversities were judged to be sufficiently distinct from potentially protective factors and appropriate given known effects of tightened restrictions as highlighted in previous studies (Perzow et al., 2021; Yip et al., 2021; Sia et al., 2022).

#### 4.2.3 Protection – social and socio-environmental factors and coping

We measured neighborhood cohesion (*α* = 0.950), by asking about, for example, “feeling a sense of belonging to the neighborhood”. Measurement of Social bonds and intimacy (*α* = 0.920) contained items like having “someone to discuss and make shared decisions with”. Both measures were composed of 7-items and rated dichotomously (true/false). For quality of relationships, we asked about being able to draw on friends, a romantic partner or spouse, and/or family and relatives for close, confiding support when needed (*α* = 0.710). Responses were measured on a 3-item, 4-point Likert scale. Harmonious living circumstances was captured by asking about people’s feelings about their living space (*α* = 0.840), e.g., “a place I am forced to remain despite being a source of stress and strife,” or “a hub of peace and tranquility” (reverse coded). This was measured using an 11-item set of questions and 5-point Likert scale, with 1 being “always or chronically” and 5 being “never.”

All above measures were self-constructed to be tailored to our study needs, many items were adapted from previously self-constructed published scales used in the Singapore context (Aw et al., 2020). As demonstrated above, all selected measures showed good internal consistency and were judged fit for purpose.

Similarly, due to the novelty of the topic a context-specific scale on COVID-19 life satisfaction (*α* = 0.820) was designed. This scale consisted of 12-item asking about how daily life and wellbeing had changed pre-and post-COVID-19. For instance, “life before COVID-19 was more complicated and less fulfilling than life nowadays.” Responses were captured on a 4-point Likert scale (i.e., 1 being “never” and 4 being “always”). Three items were removed to improve internal consistency. Negatively phrased items were reverse coded.

Coping was assessed using the validated Brief Resilience Coping Scale (BRCS; Sinclair and Wallston, 2004; Kocalevent et al., 2017) (*α* = 0.880) which captured adaptive tendencies, focusing on the effective use of coping strategies in the face of stressors. The four items consist of strategies including “looking for creative ways to alter difficult situations”; “believing that regardless of what happens, how we react is within one’s control”; “believing that one can grow in positive ways when dealing with difficult circumstances”; and “actively looking for ways to replace losses when these are encountered.” The items scored on a 5-point Likert scale (i.e., 1 being “does not describe me at all,” and 5 representing “describes me very well”).

Composite scores were collated for all scales and dichotomized at the median for use in the analysis, where 1 denoted a positive experience (e.g., strong coping), while 0 denoted a negative one (e.g., weak coping).

### 4.3 Method of analysis

Analyses were carried out using R version 1.3 for iOS.

#### 4.3.1 Adversities – capturing psychosocial circumstances that adversely affect mental health

We first tested whether the proposed adversities were associated with below average HADS. Thus we tested the differences in HADS scores between those exposed to an adversity and those not. Since distribution of scores did not meet normality, a Wilcoxon rank sum test was performed. Only adversity variables that showed statistically significant higher HADS scores (i.e., *p* value <0.05) were included in the model of the subsequent analysis. Non-parametric bootstrapping of 100,000 simulated samples was also conducted to generate the confidence intervals for the means. The cumulative effect of these adversities on HADS were then also examined. Analysis of cumulative effects helped to determine the cutoffs for “high” and “low” levels of adversity exposure, as used for analysis outlined below.

#### 4.3.2 Measuring resilient outcomes and associated characteristics

Resilient outcomes were identified as HADS scores on or below the median in the face of exposure to two or more statistically significant adversities. Conversely, vulnerable outcomes were attributed when respondents reported the same adversity exposure levels but had HADS scores above the median. The median HADS cutoff was chosen both because it maps to our theoretical definition of resilience as “doing better than expected,” and because it is known to improve the sensitivity and specificity of this measure in analysis (Bjelland et al., 2002; Löwe et al., 2004; Hansson et al., 2009).

Because adversities were selected as events that tended to reduce mental health, “resilient” outcomes were classed as achieved by those who still reached standard (on the median) or less HADS scores, and were thus seen to be managing better than expected – indeed doing well, or even exceptionally well – despite exposure to two or more adversities. The natural comparator to this would be those identified as similarly exposed but having HADS scores above the median, or “vulnerable” outcomes.

The cutoff of two adversities was chosen to capture enough adversity to warrant resilience mechanisms to be needed, and potentially kick in; this cut-off for higher adversity exposure is later verified empirically. Crosstabulations and chi-square tests comparing social and coping characteristic across resilient and vulnerable outcomes are reported.

#### 4.3.3 Examining attributes that attenuate the negative effects of adversity

To identify which individual and social variables had the potential to attenuate effects of higher adversity on mental health, logistic regression was carried out with depression and anxiety scores from HADS as the dichotomized dependent variables. Depression and anxiety scores were coded as 0 denoting presence of depression or anxiety and 1 denoting absence of depression or anxiety. This next step in resilience analysis consisted of comparing social and socio-environmental factors and coping across stratified sub-groups of those exposed to less versus more adversity. The sub-group with no or one adversity, henceforth is simply referred to as the “low adversity” group and “higher adversity” refers to the sub-group having two or more of these. All models were adjusted for age, gender, race, relationship status, work status, education level, housing type and income.

There are three key relationships of interest to be identified by this proposed analysis. Firstly, should a predictor be associated with improving mental health in both low and higher exposure groups with no statistically significant difference, or interaction effect, between the groups we can conclude that the protective effect is independent of adversity. However, if there is a statistically significant difference in improving mental health outcomes in those exposed to higher levels of adversity compared to lower levels, the predictor can be seen as promoting resilience. Lastly, if the reverse is found this indicates a weak protective effect, which will only be useful when low levels of adversity are being experienced. To identify differences in protective effects between the levels of adversity exposure, the testing of the interaction between each predictor at the low and higher levels of adversity exposure was performed.

## 5 Results

### 5.1 Population sample

Participants’ ages ranged from 21 to 81 years, with the mean age being 47 years. More than half the sample were either female (*N* = 830, 60.9%), married or co-habiting (*N* = 749, 54.9%), university graduates (*N* = 788, 57.8%) or living in a 4 to 5-room flat (*N* = 785, 57.6%). About a quarter of respondents would be considered essential workers (*N* = 181, 13.3%), with less than half the sample acquiring a monthly household income of more than SGD$9,000 (*N* = 554, 40.6%). When compared to Singapore’s 2020 census data (Department of Statistics Singapore, 2021), a bias was found for ethnicity, whereby 89.1% of the study sample were Chinese compared to 74.3% in the census data. All other sociodemographic factors were comparable with the population census data (Department of Statistics Singapore, 2021, 2023). A descriptive breakdown of the sample can be found in Supplementary Annex 2.

### 5.2 What typifies psychosocial adversity?

**Figure 2:**
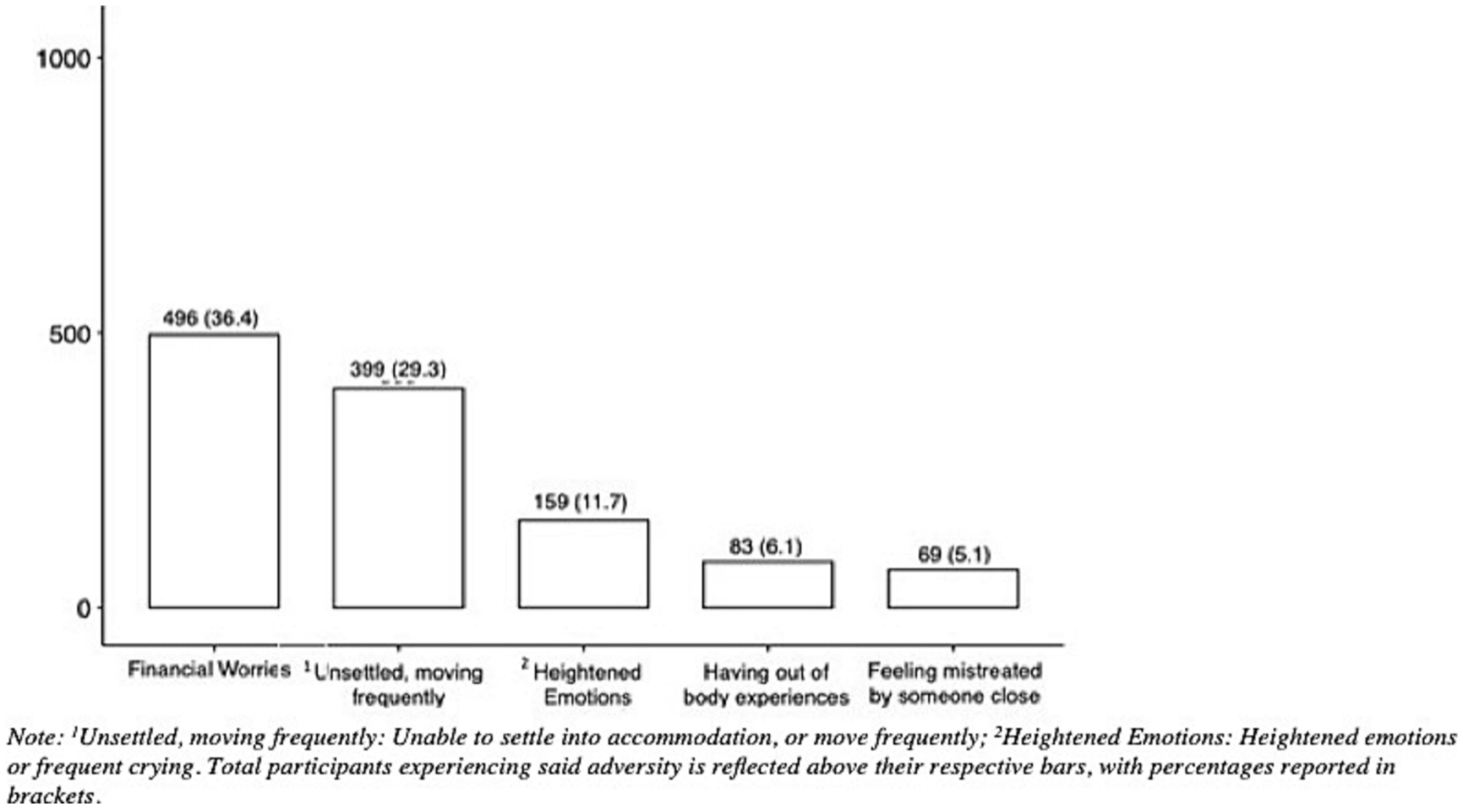
Prevalence of adversities. ^1^Unsettled, moving frequently: Unable to settle info accommodation, or move frequently; ^2^Heightened emotions: Heightened emotions or frequent crying. Total participants experiencing said adversity is reflected above their respective bars, with percentages reported in brackets.

Selected potential adversities varied in prevalence ranging from 5.1 to 36.4%, with 761 (55.8%) reporting at least one of these (see Figure 2). Table 1 shows the differences in mean HADS scores for depression and anxiety when comparing those exposed to each of the adversities to those not exposed. All five proposed adversities showed statistically significant associations with having worse mental health outcomes (higher HADS scores), and thus were included in subsequent analyses. For both depression and anxiety, the maximum increase occurred in those experiencing heightened emotions (+4.562 points and + 5.286 points respectively). The smallest increase for depression was in having to move frequently or not being able to settle into accommodation (+2.213 points); for anxiety, this was having financial worries (+2.526 points).

**Table 1:** Differences in mean for HADS scores when comparing those exposed to each of the adversities to those unexposed.

Adversities	Depression (*N* = 1,364)				Anxiety (*N* = 1,364)			
	Exposed to adversity	Unexposed to adversity	Significance for difference in mean		Exposed to adversity	Unexposed to adversity	Significance for difference in mean	
	Mean (SD)	Mean (SD)	95% CI	value of *p*	Mean (SD)	Mean (SD)	95% CI	value of *p*
^1^Heightened emotions	10.818 (4.115)	6.256 (3.867)	3.892–5.234	<0.001^***^	12.151 (4.086)	6.865 (3.434)	4.628–5.944	<0.001^***^
Having out of body experiences	10.928 (4.946)	6.519 (3.961)	3.324–5.495	<0.001^***^	11.156 (4.608)	7.243 (3.732)	2.912–4.927	<0.001^***^
^2^Mistreated/abused	10.043 (4.431)	6.614 (4.076)	2.379–4.504	<0.001^***^	11.217 (4.362)	7.282 (3.776)	2.892–4.971	<0.001^***^
Financial worries	8.369 (4.289)	5.884 (3.804)	2.031–2.941	<0.001^***^	9.089 (3.904)	6.562 (3.594)	2.109–2.946	<0.001^***^
^3^Unsettled, moving frequently	8.353 (4.216)	6.140 (3.964)	1.734–2.697	<0.001^***^	9.634 (4.059)	6.591 (3.467)	2.592–3.498	<0.001^***^

^1^Heightened emotions, Heightened emotions and frequent crying; ^2^Mistreated/abused, Feeling mistreated by someone close; ^3^Unsettled, moving frequently, Unable to settle into accommodation, or moving frequently. *P*-values are based on two sample *t*-test. ^*^p < 0.05; ^**^p < 0.01;^***^p < 0.001.

**Figure 3:**
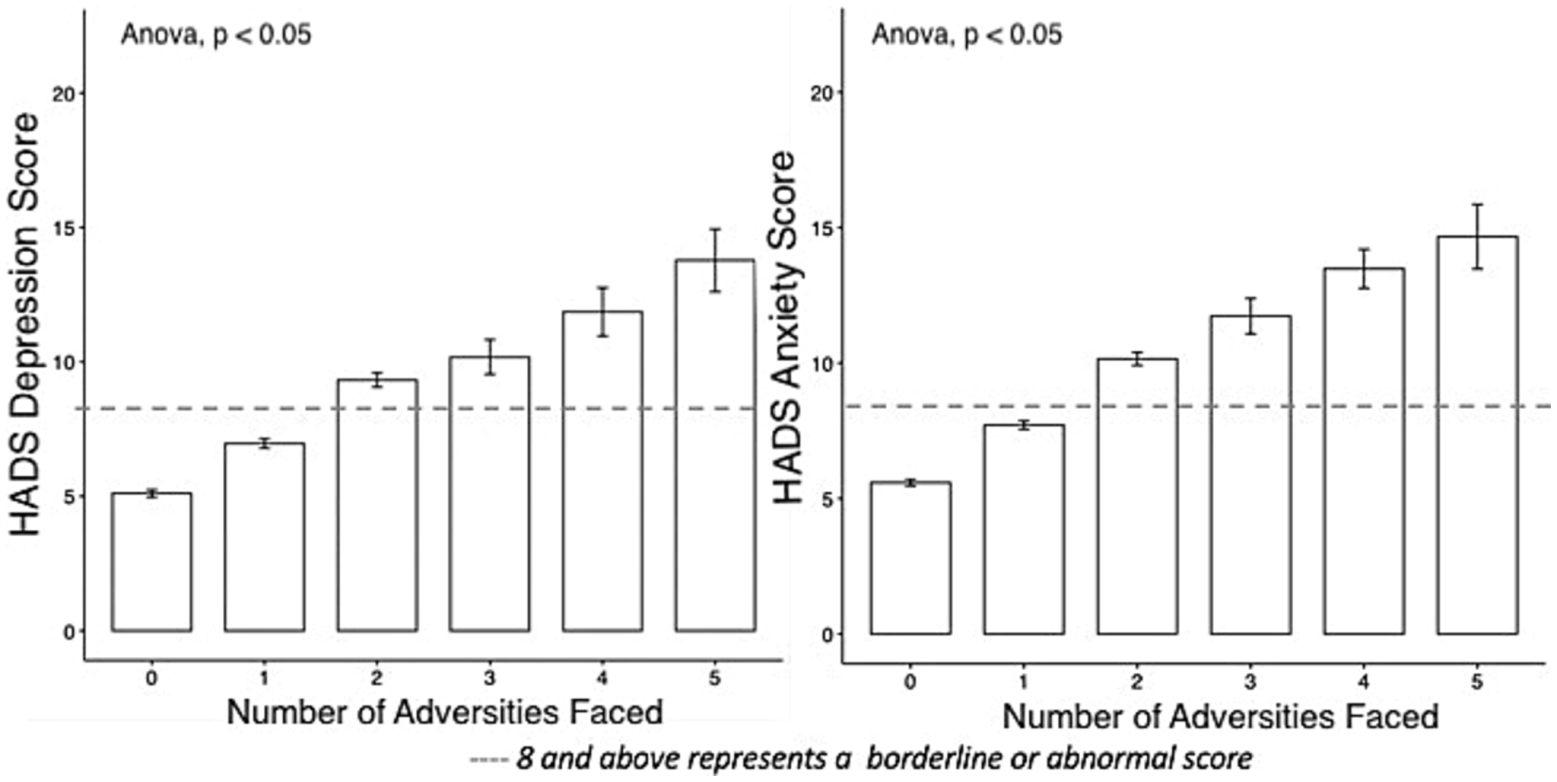
Mental health (HADS scores) in the presence of mounting adversity.

Analysis of the combined effects of adversity showed that as the number of adversities increased so did the effects on HADS (see Figure 3). In the presence of two or more adversities, the mean HADS score for depression and anxiety rose above the borderline abnormal benchmark of 8. This provided support for identification of resilient outcomes as being present when facing two or more adversity exposures yet still being able to achieve better than expected mental health outcomes. Reported HADS scores ranged from 0 to 21 both for depression and anxiety, the median and means were, respectively, 5 and 6, SD = 4.16, for depression, and both the median and mean were 6 with a SD = 3.90 for anxiety. Histograms for HADS are reported in Supplementary Annex 2, see Supplementary Figures S1, S2.

### 5.3 Who are the resilient?

Descriptive statistics for potentially protective social and socio-environmental factors and coping are reported in Supplementary Annex 2, Supplementary Tables S2, S3. *N* = 304 (or 22.3%) of the total sample were exposed to at least two adversities and qualified for analysis to identify the characteristics of the resilient versus vulnerable groups. Among the 304 participants, for depression 66 (21.8%) were classified as resilient, and the remainder 238 (78.3%) were classified as vulnerable. Similarly, for anxiety, 60 (19.7%) were classified as resilient, and 244 (80.3%) as vulnerable.

The sociodemographic profile of the resilient and vulnerable groups tended to be similar (see Table 2). With the exception of education level for depression outcomes, whereby the vulnerable (63.4%) were significantly (*χ*^2^ = 5.407, *p* = 0.040) more educated (i.e., University/Postgraduate) as compared to the resilient (48.5%). While for the anxiety outcomes, the only difference is age, whereby the vulnerable (42.2%) were found to be significantly (*χ*^2^ = 4.256, *p* = 0.039) younger, i.e., under 35 years, as compared to the resilient (26.7%).

**Table 2:** Descriptive statistics of sociodemographic factors in the resilient and vulnerable groups.

Sociodemographic factors	Depression (*N* = 304)			Anxiety (*N* = 304)		
	Resilient (*N* = 66) counts (%)	Vulnerable (*N* = 238) counts (%)	value of *p*	Resilient (*N* = 60) counts (%)	Vulnerable (*N* = 244) counts (%)	value of *p*
Age; Under 35 years old	23 (34.8)	96 (40.3)	0.506	16 (26.7)	103 (42.2)	0.039^*^
Gender; Female	40 (60.6)	141 (59.2)	0.954	30 (50.0)	151 (61.9)	0.125
Race; Chinese	54 (81.8)	209 (87.8)	0.290	51 (85.0)	212 (86.9)	0.863
Relationship status; Married or co-habiting	29 (43.9)	109 (45.8)	0.898	29 (48.3)	109 (44.7)	0.715
Work Status; Essential	24 (36.4)	106 (44.5)	0.295	25 (41.7)	105 (43.0)	0.963
Education level; University/Postgraduate	32 (48.5)	151 (63.4)	0.040^*^	30 (50.0)	153 (62.7)	0.098
Housing Type; 4 to 5-room HDB	44 (66.7)	135 (56.7)	0.190	37 (61.7)	142 (58.2)	0.732
Income; more than $9,000	25 (37.9)	104 (43.7)	0.481	26 (43.3)	103 (42.2)	0.991

*P*-values are based on chi-square test of independence. ^*^p < 0.05; ^**^p < 0.01; ^***^p < 0.001.

Generally, many significant differences were found between the resilient and vulnerable in relation to social and socio-environmental factors (see Table 3). For depression, all factors including neighborhood cohesion, quality if relationships, social bonds and intimacy, harmonious living and COVID-19 life satisfaction were found to be characteristic of the resilient group and significant differences observed. For anxiety, similar relationships were seen, with the exception of quality of relationships which was not significant (resilient 95% and vulnerable 88.5%, *χ*^2^ = 1.555, *p* = 0.212). Neighborhood cohesion was the least common form of protection. Having social bonds and intimacy as well as harmonious living circumstances were of particular interest with respect to large difference in counts between resilient and vulnerable groups.

**Table 3:** Prevalence of social and socio-environmental factors in the resilient and vulnerable groups.

Social and socio-environmental factors	Depression (*N* = 304)			Anxiety (*N* = 304)		
	Resilient (*N* = 66) count (%)	Vulnerable (*N* = 238) count (%)	value of *p*	Resilient (*N* = 60) count (%)	Vulnerable (*N* = 244) count (%)	value of *p*
Neighborhood cohesion; Composite score of more than 4	31 (47.0)	70 (29.4)	0.011^*^	28 (46.7)	73 (29.9)	0.021^*^
Quality of relationship; Composite score of more than 6	65 (98.5)	208 (87.4)	0.016^*^	57 (95.0)	216 (88.5)	0.212
Social bonds and intimacy; Composite score of more than 4	59 (89.4)	151 (63.4)	<0.001^***^	51 (85.0)	159 (65.2)	0.005^**^
Harmonious living circumstances; Composite score of more than 24	39 (59.1)	41 (17.2)	<0.001^***^	28 (46.7)	52 (21.3)	<0.001^***^
COVID-19 life satisfaction score; Composite score of more than 24	51 (77.3)	78 (32.8)	<0.001^***^	41 (68.3)	88 (36.1)	<0.001^***^

*P*-values are based on chi-square test of independence. ^*^p < 0.05; ^**^p < 0.01; ^***^p < 0.001.

Solution-driven coping strategies underpinned resilient outcome, both for depression and anxiety (see Table 4). All coping styles were thus positively connected to depression outcomes. For Anxiety this was the case with the exception for seeking creative alternatives and when the Brief Resilience Coping Scale was used as a composite score. In addition, when it came to anxiety all coping items showed less magnitude of effect. The biggest magnitude of difference was found for believing “that dealing with difficult situations can lead to positive growth” when accounting for analysis of both depression (resilient 89.4% and vulnerable 46.6%, *χ*^2^ = 36.605, *p* < 0.001) and anxiety (resilient 85% and vulnerable 48.8%, *χ*^2^ = 24.195, *p* < 0.001).

**Table 4:** Prevalence of coping strategies in the resilient and vulnerable groups.

Coping strategies	Depression (*N* = 304)			Anxiety (*N* = 304)		
	Resilient (*N* = 66) count (%)	Vulnerable (*N* = 238) count (%)	value of *p*	Resilient (*N* = 60) count (%)	Vulnerable (*N* = 244) count (%)	value of *p*
Searching for creative ways to alter difficult situations; Score of more than 4	44 (66.7)	98 (41.2)	<0.001^***^	35 (58.3)	107 (43.9)	0.062
Believes that their reaction toward a situation is within their control; Score of more than 4	47 (71.2)	97 (40.8)	<0.001^***^	37 (61.7)	107 (43.9)	0.02^*^
Believes that dealing with difficult situations can lead to growth in positive ways; Score of more than 4	59 (89.4)	111 (46.6)	<0.001^***^	51 (85.0)	119 (48.8)	<0.001^***^
Actively looking for ways to replace losses encountered in life; Score of more than 4	48 (72.7)	96 (40.3)	< 0.001^***^	39 (65.0)	105 (43.0)	0.004^**^
Brief resilience coping scale; Composite score of more than 15	49 (74.2)	112 (47.1)	<0.001^***^	38 (63.3)	123 (50.4)	0.098

*P*-values are based on chi-square test of independence. ^*^p < 0.05; ^**^p < 0.01; ^***^p < 0.001.

### 5.4 How are the negative effects of adversity attenuated?

Analyses examining factors that attenuate the negative impact of adversity are based on *N* = 304 for higher exposure to adversity and *N* = 1,060 for low exposure to adversity. Statistical significance was found for most of the social and socio-environmental as well as coping predictors in both low and higher adversity subgroups. However, only three predictors had significant interactions, indicating a difference in protective effect between low and higher adversity subgroups (see Table 5).

**Table 5:** Social and socio-environmental factors and coping related strategies that confer protective effects during exposure to low versus higher adversity.

Social and socio-environmental factors	Depression					Anxiety				
	Low adversity *N* = 1,060		Higher adversity *N* = 304		Interaction effect	Low adversity *N* = 1,060		Higher adversity *N* = 304		Interaction effect
	Odds ratio (95% CI)	value of *p*	Odds ratio (95% CI)	value of *p*	value of *p*	Odds ratio (95% CI)	value of *p*	Odds ratio (95% CI)	value of *p*	value of *p*
Neighborhood cohesion	1.929 (1.469–2.544)	<0.001^***^	1.981 (1.114–3.517)	0.019^*^	0.651	1.573 (1.186–2.097)	0.002^**^	1.902 (1.047–3.446)	0.034^*^	0.453
Quality of relationship	2.858 (2.006–4.101)	<0.001^***^	11.231 (2.246–204.596)	0.020^*^	0.152	1.916 (1.346–2.726)	<0.001^***^	2.867 (0.921–12.697)	0.104	0.010^*^
Social bonds and Intimacy	2.116 (1.627–2.759)	<0.001^***^	5.707 (2.578–14.558)	<0.001^***^	0.411	1.517 (1.158–1.992)	0.003^**^	3.304 (1.565–7.702)	0.003^**^	0.472
Harmonious living circumstances	4.182 (3.203–5.482)	<0.001^***^	8.998 (4.699–17.868)	<0.001^***^	0.035^*^	3.400 (2.592–4.476)	<0.001^***^	4.358 (2.269–8.520)	<0.001^***^	0.794
COVID-19 life satisfaction score	7.237 (5.395–9.807)	<0.001^***^	6.785 (3.613–13.409)	<0.001^***^	0.430	4.544 (3.391–6.146)	<0.001^***^	3.731 (2.018–7.119)	<0.001^***^	0.983
Coping strategies										
Searching for creative ways to alter difficult situations	2.588 (2.007–3.347)	<0.001^***^	3.283 (1.824–6.084)	<0.001^***^	0.709	1.541 (1.189–2.000)	0.001^**^	2.022 (1.121–3.709)	0.021^*^	0.628
Believes that their reaction toward a situation is within their control	3.183 (2.456–4.136)	<0.001^***^	4.129 (2.252–7.869)	<0.001^***^	0.655	2.606 (2.000–3.402)	<0.001^***^	2.150 (1.190–3.958)	0.012^*^	0.449
Believes that dealing with difficult situations can lead to growth in positive ways	3.481 (2.637–4.614)	<0.001^***^	12.162 (5.486–31.150)	<0.001^***^	0.013^*^	2.332 (1.767–3.080)	<0.001^***^	7.461 (3.553–17.380)	<0.001^***^	0.015^*^
Actively looking for ways to replace losses encountered in life	2.331 (1.811–3.008)	<0.001^***^	4.220 (2.316–7.995)	<0.001^***^	0.112	1.575 (1.216–2.043)	0.001^**^	2.590 (1.430–4.808)	0.002^**^	0.181
Brief resilience coping scale	2.523 (1.956–3.261)	<0.001^***^	3.860 (2.082–7.492)	<0.001^***^	0.424	1.761 (1.356–2.287)	<0.001^***^	1.835 (1.013–3.397)	0.049^*^	0.858

Low adversity refers to exposure to 0 or 1 adversity, while higher adversity refers to exposure to 2 or more adversities. The model is adjusted for age, gender, race, relationship status, work status, education level, housing type, and income. Interaction effect corresponds to the interaction term between a factor and the level of adversity which is included into the model as a predictor together with the factor, level of adversity, age, gender, race, relationship status, education level, housing type, and income (*N* = 1,364). ^*^p < 0.05; ^**^p < 0.01; ^***^p < 0.001.

For the social and socio-environmental factors these included harmonious living circumstances, with depression as the outcome (interaction *p* = 0.035). Harmonious living circumstances conferred protection when faced with low exposure to adversity (OR = 4.182; 95% CI 3.203–5.482), but also increased in effect with higher exposure to adversity (OR = 8.998; 95% CI 4.699–17.868). On the other hand, a different interaction effect (interaction *p* = 0.010) was found for quality of relationships with anxiety as the outcome. Quality of relationships was only significantly protective at low levels of adversity exposure (OR = 1.916; 95% CI 1.346–2.726; *p* < 0.001) and ceased to be protective at higher levels (OR = 2.867; 95% CI 0.921–12.697; *p* = 0.104).

For coping, a resilient effect was observed for the ability to harness the belief that difficult situations can lead to growth in both depression (interaction *p* = 0.013) and anxiety (interaction *p* = 0.015). Specifically, for depression in low levels of adversity (OR = 3.481; 95% CI 2.637–4.614), the protective effect was smaller in comparison to higher levels of adversity (OR = 12.162; 95% CI 5.486–31.150). A similar phenomenon was observed for anxiety, which showed those exposed to higher levels of adversity experienced greater protective effects (OR = 7.461; 95% CI 3.553–17.380) in comparison to the less exposed (OR = 2.332; 95% CI 1.767–3.080).

Additionally, to investigate the effects of skewness in some variables (i.e., neighborhood cohesion and quality of relationships), we undertook sensitivity analyses, and reran analyses in these by changing the threshold to dichotomize predictors at the mean rather than the median, as well as by changing the threshold to include the threshold value. Findings were generally consistent with the reported results, though, especially for high levels of adversity, the magnitude of the odds ratios were attenuated, and a loss of significance was detected for neighborhood cohesion at high levels of adversity. Sensitivity analysis generally showed the results to be stable, though to treat findings on neighborhood cohesion with some caution.

## 6 Discussion

In this paper we consider effects of community restrictions on mental health and the role of risk and resilience. This was carried out by examining mental health, adversity and protective exposures during Singapore’s Phase 2 Heightened Alert. Resilience, as we defined it, was observed in around 20% of those exposed to the selected adversities. Adversities were identified both from existing literature (Xiong et al., 2020; Castellano-Tejedor et al., 2022) and empirically verified in the present analyses. Our study has also demonstrated that for significant effects to present on mental health outcomes multiple adversities will need to be detected, and that as adversities mount so can their effects on mental health. Our findings echo longstanding traditions of resilience research (Werner and Smith, 1979; Werner and Smith, 1982; Masten, 2001; Gallo et al., 2009; Keyes, 2009; Masten, 2015; Hildon et al., 2018; Chan et al., 2022; Zhang et al., 2022) and support the central hypothesis that risk is cumulative and exposure to minimal amounts of adversity can be insufficient to trigger certain protective mechanisms (Cicchetti, 2010; Malhi et al., 2019).

Our work also demonstrates the notion of the Loads-Levers-Lifts model, which acknowledges that protection itself is multi-faceted and merits to be studied in relation to specific contexts and degrees of adversity exposure. Correspondingly during periods of intermittent community restrictions and lockdown, the present analysis showed that is catalyzed mental health Loads, measured as cumulative adversities herein. It was demonstrated that harmonious living situations will Lift and offset higher levels of adversity and help enable resilience to depression. In addition, coping by believing that dealing with difficult situations can lead to growth in positive ways shown to be central to Lifting outcomes related both to depression and anxiety. Those with resilient outcomes were found to be more likely to have access to these and other forms of protection. Notably, aligning with the alternative model of existential positive psychology (Wong P. T. P. et al., 2021), where adversity is understood as an important element that allows people to ultimately flourish. Indeed, long-term wellbeing is attained through the understanding that suffering will occur, but also a myriad of ways of overcoming it will be present. Therefore, our study proposes that Levers, such as proactive therapeutic initiatives that enable resilience should tap into this mode of coping. For instance, building resilient capabilities, cultivating inner agency, and addressing anxiety and feelings of uncertainty through Cognitive Behavioral Therapy (CBT; Wong et al., 2006; Lusk and Meinyk, 2013; Van Orden et al., 2021). Furthermore, resilience informed counseling and social work should plan to leverage a focus on achieving better harmony at home and in relationships.

During periods such as Singapore’s Phase 2 Heightened Alert, where movement restrictions are being applied intermittently, above mentionned interventions should be adaptable. These can be enabled in person, if restrictions allow, but also virtually through digital platforms when not. Evidence has shown that CBT delivered online can aid in mitigating health problems (Cuijpers et al., 2008) and that digital platforms can effectively treat depression and anxiety during COVID-19 (Aminoff et al., 2021; Mahoney et al., 2021; Song et al., 2021). Moreover, digital channels have been shown to be critical in aiding people to maintain a “sense of connectedness” to sustain relationship with their loved ones, providing the means for people to maintain and repair relationships during the pandemic (Genc et al., 2022) and beyond. More generally, it is important to note that since resilience will hinge on avoiding multiple adversities (Peek, 2008; Hildon et al., 2010) as well as acquiring sufficient protective buffering to tip the scales back into one’s favor if needed, *any* intervention that seeks to engage these mechanisms (Cho et al., 2022; Marciano et al., 2022; Silveira et al., 2022; Waters et al., 2022) will be invoking resilient processes.

Our study has its limitations. For instance, while we were able to detect many protective factors more generally, detecting those specific to higher levels of adversity was limited by low power, and some skewness in the data. However, we were still able to capture clear and consistent evidence of resilient characteristics in sub-group analysis as well as some stable interaction effects relating to higher exposure to adversity and corresponding lifts. That said, our present analysis is unable to drill down into those unaffected by COVID-19 restrictions, for example “hardy” personality types (Kobasa et al., 1982; Florian et al., 1995; Magai et al., 2003) or chart bouncing back (Netuveli et al., 2008) over time as such distinctions are best analyzed longitudinally, and we used cross-sectional data. Moreover, quantitative approaches do not allow for reflexive and retrospective analysis, qualitative approaches designed to reduce recall bias and explore lived experiences need to be used to address these issues.

Nevertheless, the current results provide insights into the mental fatigue faced by the community after undergoing multiple waves of COVID-19 restrictions. Future studies are needed to better explore and use longitudinal approaches to capture long-term effects, and fluctuations of living with COVID-19 over time.

## 7 Conclusion

In conclusion, early identification of loads, i.e., cumulative adversities which burden mental health and lifts, i.e., protective factors which offset these is fundamental to addressing the effects of the pandemic on our communities. It is also crucial that theory and methods of studying and engaging in resilience become mainstream. The importance of this phenomenon should be emphasized both to public health practitioners and the public as we navigate unprecedented social changes in how we relate and interact.

## Data availability statement

The data presented in this article are not readily available due to the sensitive nature of the study, and to preserve anonymity of the participants, as per the study team’s commitment during institutional ethical review. Requests to access the datasets should be directed to Zhildon@nus.edu.sg.

## Ethics statement

This study involved humans and was approved by the National Healthcare Group Domain Specific Review Board, Singapore (Reference No. is 2018/01203). The study was conducted in accordance with the local legislation and institutional requirements. The participants provided their written informed consent to take part.

## Author contributions

ZJ-LH conceptualized the study design. AYC, AMSES, MI-CC, and ZJ-LH contributed to the data collection. AYC conducted the data analysis under the guidance of CST. AYC drafted the manuscript with contributions from FJHC and editorial from ZJ-LH. All authors contributed to editing the manuscript and approved the publication.
