# Supplementary material for: Examining resilience in Singapore in the face of COVID-19 community restrictions
Source: Front Psychol. 2023 Nov 29;14:1082148. doi: 10.3389/fpsyg.2023.1082148 (PMC10716300; doi:10.3389/fpsyg.2023.1082148)
Supplement: Supplementary file 1 [file Data_Sheet_1.docx]

Supplementary Materials

*
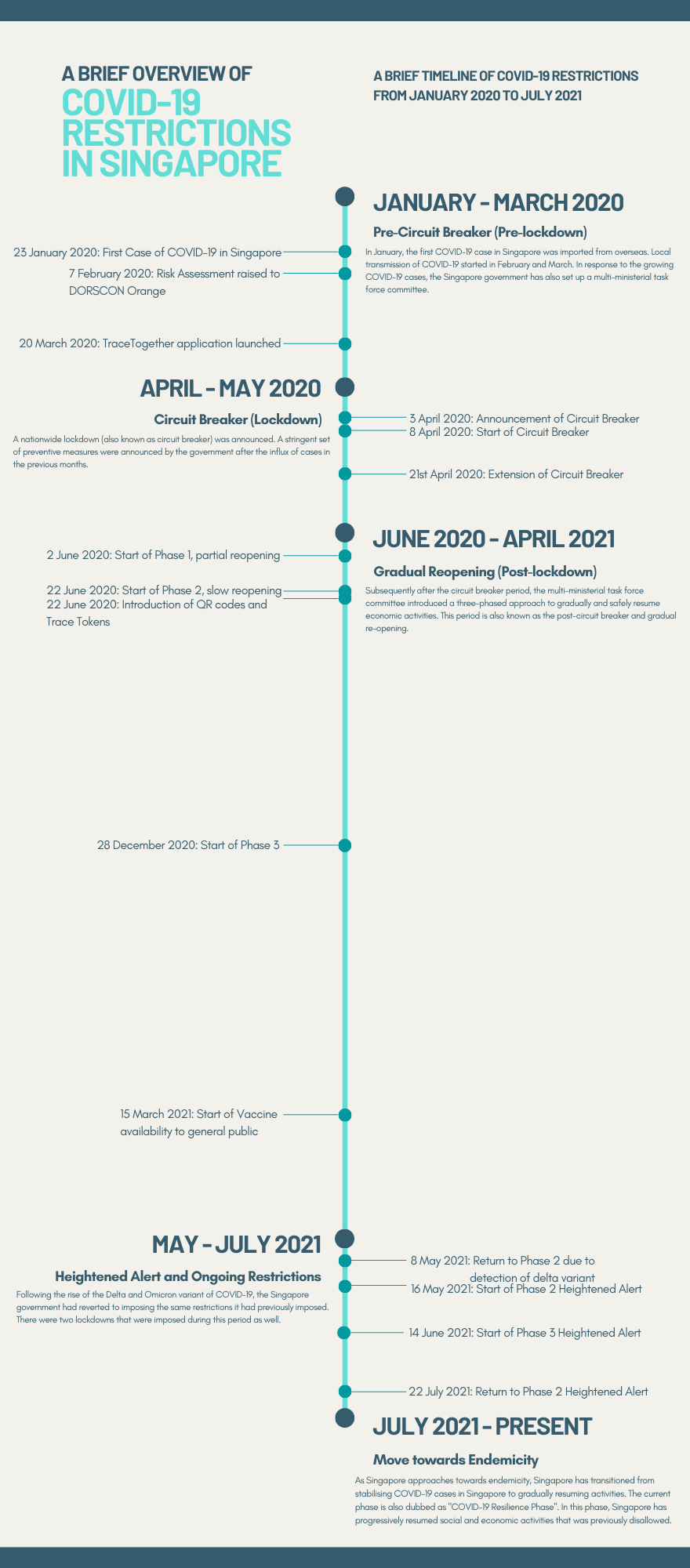
*Supplementary Annex 1

*Infographic S1: COVID-19 Community Restrictions Timeline in Singapore*

**Supplementary Annex 2 (Tables S1-S3 and Figures S1&2)**

Descriptive statistics for variables relating to sociodemographic factors; mental health outcomes; social networks, and coping strategies (N=1364)

*Table S1: Descriptive Statistics for Sociodemographic Variables*

| Sociodemographic Variables | Total Sample (N=1364) |
| --- | --- |
|  | N (%) |
| Age; Under 35 years old | 356 (26.1) |
| Gender; Female | 830 (60.9) |
| Race; Chinese | 1216 (89.1) |
| Relationship Status; Married or co-habiting | 749 (54.9) |
| Work Status; Essential | 181 (13.3) |
| Education level; University/Postgraduate | 788 (57.8) |
| Housing Type; 4 to 5-room HDB | 785 (57.6) |
| Income; more than $9,000 | 554 (40.6) |

*Note. HDB is an abbreviation for Housing Development Board flats.*

*Figure S1. Histogram for Hospital Anxiety and Depression Scale (HADS) Depression Scores*

**

*Figure S2. Histogram for Hospital Anxiety and Depression Scale (HADS) Anxiety Scores*

*Table S2: Descriptive Statistics for Social Networks*

| Social Networks and Cohesion | Total Sample (N=1364) | | |
| --- | --- | --- | --- |
|  | N (%) | Mean | Range |
| Neighbourhood Cohesion; Composite score of more than 4 | 528 (38.7) | 2.0 | 0 to 7 |
| Quality of Relationship; Composite score of more than 6 | 1295 (94.9) | 9.4 | 3 to 12 |
| Social Bonds and Intimacy; Composite score of more than 4 | 1047 (76.8) | 5.0 | 0 to 7 |
| Harmonious Living Circumstances; Composite score of more than 24 | 740 (54.3) | 32.9 | 14 to 64 |
| COVID-19 Life Satisfaction Score; Composite score of more than 24 | 825 (60.5) | 29.5 | 12 to 48 |

*Table S3: Descriptive Statistics for Coping Strategies*

| Coping Strategies | Total Sample (N=1364) | | |
| --- | --- | --- | --- |
|  | N (%) | Mean | Range |
| Searching for creative ways to alter difficult situations; Score of more than 4 | 695 (51.0) | 3.5 | 1 to 5 |
| Believes that their reaction towards a situation is within their control; Score of more than 4 | 785 (57.6) | 3.6 | 1 to 5 |
| Believes that dealing with difficult situations can lead to growth in positive ways; Score of more than 4 | 920 (67.4) | 3.7 | 1 to 5 |
| Actively looking for ways to replace losses encountered in life; Score of more than 4 | 698 (51.2) | 3.5 | 1 to 5 |
| Brief Resilience Coping Scale; Composite score of more than 15 | 764 (56.0) | 14.2 | 4 to 20 |
